# Improvement of clinical quality indicators through reorganization of the acute care by establishing an emergency department-a register study based on data from national indicators

**DOI:** 10.1186/s13049-014-0060-4

**Published:** 2014-11-05

**Authors:** Maria Søe Mattsson, Nick Mattsson, Hanne B Jørsboe

**Affiliations:** Faculty of Health Science, University of Southern Denmark, 5230 Odense M, Denmark; Emergency Department, Hospital of Nykøbing Falster, 4800 Nykøbing Falster, Denmark; Department of Cardiology, Bispebjerg Hospital, 2400 Copenhagen, NV Denmark

**Keywords:** Emergency Department, Reorganization, Indicators, Mortality

## Abstract

**Background:**

The Emergency Departments (EDs) reorganization process in Denmark began in 2007 and includes creating a single entrance for all emergency patients, establishing triage, having a specialist in the front and introducing the use of electronic overview boards and electronic patient files. The aim of this study was to investigate the quality of acute care in a re-organized ED based on national indicator project data in a pre and post reorganizational setting.

**Methods:**

Quasi experimental design was used to examine the effect of the health care quality in relation to the reorganization of an ED. Patients admitted at Nykøbing Falster Hospital in 2008 or 2012 were included in the study and data reports from the national databases (RKKP) regarding stroke, COPD, heart failure, bleeding and perforated ulcer or hip fracture were analysed. Holbæk Hospital works as a control hospital.

Chi-square test was used for analysing significant differences from pre-and post intervention and Z-test to compare the experimental groups to the control group (HOL). P < 0.05 was considered statistically significant.

**Results:**

We assessed 4584 patient cases from RKKP. A significant positive change was seen in all of the additional eight indicators related to stroke at NFS (P < 0.001); however, COPD indicators were unchanged in both hospitals. In NFS two of eight heart failure indicators were significantly improved after the reorganization (p < 0.01). In patients admitted with a bleeding ulcer 2 of 5 indicators were significantly improved after the reorganization in NFS and HOL (p < 0.01). Both compared hospitals showed significant improvements in the two indicators concerning hip fracture (p < 0.001). Significant reductions in the 30 day-mortality in patients admitted with stroke were seen when the pre- and the post-intervention data were compared for both NFS and HOL (p = 0.024).

**Conclusions:**

During the organisation of the new EDs, several of the indicators improved and the overall 30 days mortality decreased in the five diseases. The development of a common set of indicators for monitoring acute treatment at EDs in Denmark is recommended.

## Background

In Denmark the choice was made to reorganise the acute care by the establishment of Emergency Departments with observation units. The aim of the new EDs was to improve the quality of the diagnostic process and allow for an earlier diagnosis and treatment of all types of acute patients based on international experience [1]. Emergency Medicine (EM) as a discipline has existed for more than 40 years in the USA and has served as a model for international experience in this field as well as the interventions in this study [2].

The international experiences have shown that the quality of treatment in EDs can be improved by using triage [3-6], optimising the flow of patients into and out of the ED [3,7-9], optimising teamwork [4] and by the introduction of a fast-track diagnostic workup for patients with less severe symptoms [9].

A strong tradition of monitoring the health care services and quality of health care exits in Denmark; however, there are currently no general accepted national quality indicators for the acute treatment of patients [10]. Although a few clinical databases related to Emergency Medicine were under development in 2013 [11,12] the usual quality measurements of choice have been to monitor different time intervals, e.g. door to treatment, which are easy accessible data for administrative use. These intervals, however, does not differentiate properly between the different acute care needs of patients.

The aim of this study is to investigate the impact of reorganization of the acute care in a new Emergency Department with observation units on the quality of health care, including mortality rate, as monitored by five selective acute conditions; stroke, acute gastrointestinal bleeding and perforation, heart failure, COPD, COPD with pneumonia and hip fractures.

## Methods

### Study design and setting

**Figure 1:**
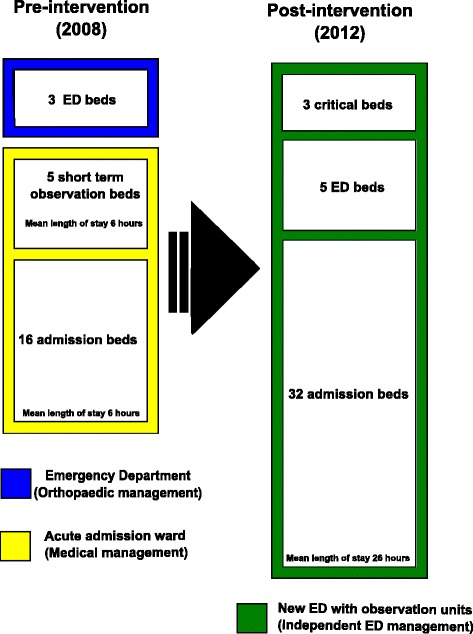
**Organizational change at Nykøbing Hospital.**

A quasi experimental design was used, including both pre- (baseline) and post-intervention data. The uniform package of interventions constituted several elements, including the following: an enhanced focus on improved admission, early stabilisation and treatment of patients through the implementation of a triage system, earlier bedside assessment of patients by nurses, an increased availability of senior doctors, improved competence of the entire staff and the implementation of electronic white boards and patient files to increase the focus on patient safety. Furthermore, a specific protocol for stroke patients served as a tool for optimising the treatment of patients and was used in the Hospital of Nykøbing Falster (NFS) as an applicable model for the treatment of acute diseases in general. The organizational changes are described in Figure 1.

Baseline data (pre-intervention) were collected from the NIP data register in the period January 2008 – December 2008 and included patients who were admitted to the ED at NFS. Post-intervention data were collected from the Region’s Clinical Quality Development Programme (RKKP) [10] from January 2012 – December 2012 and comprised patients who were admitted to the ED at the same hospital. The control group consisted of comparable patients (as defined in the national inclusion criteria) from another community hospital in Region Zealand, the ED at the Hospital of Holbæk (HOL). By using HOL as a control hospital, it provides us with the opportunity to compare the changes in the indicators, as HOL underwent the same reorganization process in the ED although organizational differences existed between the two hospitals.

NFS has an uptake area with 140.000 citizens. The activity level within the ED changed from 2008 to 2012. A reduction of 3.476 patients with small injuries was observed during this time period (24.249 in 2008; 20.773 in 2012), whereas the total number of patients admitted to the ED increased by 3.427 (12.861 in 2008; 16.288 in 2012) (OPUS: local administrative system). The choice was made not to compare each year between 2008 and 2012 as the implementation of interventions were initiated in 2009 and first completed in the year of 2011. Additionally the interventions were adjusted during the implementation process.

### Population and measurements

To measure the quality of healthcare, designated by a national board of specialists within each disease area selected a series of measures (indicators). The indicators have been selected as they are considered particularly important in the assessment of whether the quality of care is at the desired level [10]. Specific indicators for each disease were selected based on evidence of their relevance for the acute admission of patients and their potential benefits early in the patients’ pathway through the acute care process. The indicators measures either entire processes or specific outcomes. The processes represent data of examinations, treatment by physicians, treatment by other health professionals, screenings and outcome indicators represent data regarding readmissions and mortality. The RKKP (former NIP database) has expanded its scope through the years, limiting the indicators to those present in both 2008 and 2012. Because of the very few hospitalized patients with heart failure at HOL, the management of department decided to stop reporting to RKKP in 2011 and resulting in missing data in the results.

The study population consisted of acutely ill patients who sought medical attention in the ED and who met the criteria as reported to the Region’s Clinical Quality Development databases for the following diagnoses: stroke, COPD, heart failure, hip fracture and acute gastrointestinal bleeding and perforation [13-17]. All are among the 20 most common illnesses seen in an emergency care setting.

We used these data as a benchmark for critical indicators and as a quality standard in the reorganization of the EDs with pre and post analyses. As the indicators are a national requirement, data sets could be measured against comparable hospitals as well as data on a national level.

This study utilizes indicators used in all Danish hospitals over the last seven years and are all validated by the RKKP based on a clinical assessment of three main issues; does the indicator measure the clinical pathway of interest; is the indicator able to identify known variations in the quality of different health care departments considering the patient population as well and are golden standards available [10].

#### Data analysis

Frequency distributions were constructed for the datasets and interpreted using frequencies and percentages. To test for significant differences, the results were analysed using the chi-squared test. A two-proportion z-test was used to compare the experimental groups to the control group (HOL). Categories with fewer than 5 responses were folded with an adjacent category. In all analyses p < 0.05 was considered statistically significant. Data were analyzed using STATA version 11 software.

#### Ethical considerations

Upon application to RKKP we got access to the relevant databases and permission to use data in the study. Patient anonymity was protected throughout the research process. This study was approved by the hospitals of NFS and HOL as well as the Hospitals Ethics Committee and the Helsinki Declaration was complied with. The study was approved by the Danish Data Protection Agency.

## Results

We assessed 4584 patient cases from RKKP. In 2008 (pre-intervention) 1914 patient cases were included and in 2012 (post-intervention) 2670 patient cases. The mean age and gender of the participants in the pre- and post-intervention groups did not differ significantly in NFS. Fewer women with hip fractures were admitted in 2012 in HOL. Between hospitals, significant gender differences is seen for COPD in 2012 and hip-fracture both pre- and post intervention (Table 1). Changes of indicators of each of the six diagnoses from pre- to post intervention are described in the following.

**Table 1:** **Study population: Nykøbing and Holbæk Hospitals**

		**2008**			**2012**			
**Diagnoses**	**Location**	**Patients reported 2008 (n)**	**Mean age (range)**	**Gender (female) Percent**	**Patients reported 2012 (n)**	**Mean age (range)**	**Gender (female) Percent**	**P-value Proportions z-test between years at hospital level**
**Stroke**	NFS	293	72.46 (42–99)	43	212	72.63 (34–97)	48	0.133
	HOL	652	70.76 (33–102)	47	634	71.21 (34–99)	48	0.356
**COPD**	NFS	69	71.28 (38–93)	45	530	70.60 (34–94)	45*	0.490
	HOL	121	70.75 (49–89)	54	437	70.64 (30–98)	57*	0.247
**Heart failure**	NFS	136	69.90 (36–98)	31	205	70.82 (22–94)	22	0.026
	HOL	109	66.30 (19.93)	32	#			
**Bleeding ulcer**	NFS	29	71.29 (37–92)	52	104	72.14 (36–94)	44	0.237
	HOL	10	78.17 (49–88)	70	64	73.79 (45–94)	44	0.061
**Perforated ulcer**	NFS	6	65.80 (48–81)	67	16	72.50 (44–94)	25	0.035
	HOL	4	61.53(49–76)	75	8	65.23 (29–89)	50	0.203
**Hip fracture**	NFS	214	81.47 (66–101)	67*	248	82.14 (65–100)	73*	0.108
	HOL	271	84.29 (65–102)	75*	212	81.73 (65–98)	65*	0.009

*: z-test p<0.05 Gender differences between NFS and control hospital (HOL).

#Data from HOL 2012 are missing.

### Stroke

A significant positive change in all of the additional eight indicators related to stroke at NFS was seen in patients admitted with a tentative diagnosis of stroke, Table 2. At HOL 5 of 8 stroke indicators showed a positive significant change while one indicator decreased significantly; “Assessed by a physiotherapist” (pre: 94.53% vs. post: 89.73%, p = 0.002) (Table 2).

**Table 2:** **Stroke indicators**

**Indicator**	**Location**	**Pre percent (n) 2008**	**Post percent (n) 2012**	**p-value chi2-test**
Patients admitted directly/transferred within second day of hospitalization to a stroke unit?	NFS	65.86 (161/249)*	97.64 (207/212)*	<0.0001
	HOL	99.85 (649/650)*	99.21 (627/632)*	0.095
	National (mean)	89.00	94.00	
	Standard %	min. 90	min. 90	
Patients receiving antiplatelet therapy within second hospitalization days?	NFS	75.95 (120/158)*	96.89 (156/161)	<0.0001
	HOL	92.20 (402/436)*	97.01 (455/469)	0.001
	National (mean)	87.00	94.00	
	Standard %	min. 95	min. 95	
Patients receiving oral anticoagulation therapy within 14 days?	NFS	60.00 (12/20)*	100 (20/20)	0.002
	HOL	83.61 (51/61)*	96.25 (77/80)	0.010
	National (mean)	73.00	89.00	
	Standard %	min. 95	min. 95	
Patients with CT/MR scans on the day of admission?	NFS	44.85 (122/272)*	81.99 (173/211)	<0.0001
	HOL	86.97 (554/637)*	84.54 (536/634)	0.216
	National (mean)	67.00	86.00	
	Standard %	min. 80	min. 80	
Patients assessed by a physiotherapist within second hospitalization day?	NFS	68.80 (172/250)*	93.00 (186/200)	<0.0001
	HOL	94.53 (588/622)*	89.73 (524/584)	0.002
	National (mean)	73.00	88.00	
	Standard %	min. 90	min. 90	
Patients assessed by an occupational therapist within second hospitalization day?	NFS	60.64 (151/249)*	82.84 (169/204)	<0.0001
	HOL	81.28 (508/625)*	81.60 (479/587)	0.886
	National (mean)	70.00	86.00	
	Standard %	min. 90	min. 90	
Patients’ nutrition status screened within second hospitalization day?	NFS	34.00 (68/200)*	48.94 (92/188)*	0.003
	HOL	92.36 (592/641)*	97.12 (607/625)*	<0.0001
	National (mean)	68.00	84.00	
	Standard %	min. 90	min. 90	
Ultrasound/CT/MR angiography of the neck vessels within 14 days?	NFS	20.65 (32/155)	80.42 (115/143)*	<0.0001
	HOL	10.34 (42/406)	69.13 (309/447)*	<0.0001
	National (mean)	42.00	84.00	
	Standard %	min. 90	min. 90	

NFS and HOL compared to the national mean and the national standard: 2008 and 2012.

*Significant differences between hospitals compared in years (two proportions z-test).

Despite the general improvement three indicators at NFS were in post intervention data analyses still below national standard; Assessment of need for occupational therapy, nutrition screening and ultrasound/CT-/MR angiography of the neck vessels, Table 2.

### COPD

COPD indicators were unchanged comparing the pre- and post-intervention data from NFS and HOL. Although not significant, there was a decreased tendency of readmission of patients with COPD treated at NFS (25.00% vs. 18.60%, p = 0.21) making the post intervention data similar to HOL, which showed increasing readmission tendencies in the same period (14.29% vs. 18.49%, p = 0.29) (Table 3).

**Table 3:** **COPD indicators**

**Indicator**	**Location**	**Pre percent (n) 2008**	**Post percent (n) 2012**	**p-value chi2-test**
Hospitalised for acute exacerbation and receipt of NIV** treatment	NFS	4.35 (3/69)	6.60 (35/530)	0.470
	HOL	4.13 (5/119)	9.15 (40411)	0.073
	National (mean)	8.00	9.00	
	Standard %	min. 10	min.10	
Hospitalised for acute exacerbation and readmission within 30 days	NFS	25.00 (17/68)*	18.60 (93/500)	0.210
	HOL	14.29 (17/119)*	18.49 (76/411)	0.288
	National (mean)	#	18.00	
	Standard %	None	None	

NFS and HOL compared to the national mean and the national standard: 2008 and 2012.

*Significant differences between hospitals compared in years (two proportions z-test).

**NIV:” non-invasive ventilation”.

#Data stems from re-auditation.

### Heart failure

In NFS two of eight heart failure indicators were significantly improved after the reorganization: “echocardiography” (88.97% vs. 97.55%, p = 0.001) and “exercise by physiotherapist” (11.32% vs. 41.42%, p < 0.0001) while data demonstrate a significant decrease in to 2 out of 8 indicators; “NYHA classification” (96.32% vs. 90.69%, p = 0.05) and “initiated a structured training program” (92.59% vs. 84.62%, p = 0.05). Comparison to HOL is not possible because of missing data from HOL in 2012 (Table 4).

**Table 4:** **Heart failure indicators**

**Indicator**	**Location**	**Pre percent (n) 2008**	**Post percent (n) 2012**	**p-value chi2-test**
Echocardiography	NFS	88.97 (121/136)	21.57 (44/204)	0.001
	HOL	100 (109/109)	#	
	National (mean)	87.00	94.00	
	Standard %	min.90	min.90	
NYHA classification	NFS	96.32 (131/136)*	90.69 (185/204)	0.047
	HOL	100 (109/109)*	#	
	National (mean)	74.00	92.00	
	Standard %	min.90	min.90	
Started or attempted treatment with ACE inhibitor/ATII-receptor antagonist? (only patients with impaired systolic function)	NFS	90.99 (101/111)*	89.94 (152/169)	0.771
	HOL	100 (89/89)*	#	
	National (mean)	83.00	92.00	
	Standard %	min.90	min.90	
Started or attempted treatment with beta blockers (only patients with impaired systolic function)	NFS	88.18 (97/110)	84.02 (142/169)	0.333
	HOL	90.91 (80/88)	#	
	National (mean)	72.00	88.00	
	Standard %	min.80	min.80	
Started or attempted treatment with aldosterone antagonist (only patients with impaired systolic function)	NFS	39.78 (37/93)*	48.44 (62/128)	0.202
	HOL	20.00 (14/70)*	#	
	National (mean)	25.00	36.00	
	Standard %	min.35	min.35	
Referred to physical exercise by physiotherapist	NFS	11.32 (12/106)	41.42 (70/169)	<0.0001
	HOL	10.23 (9/88)	#	
	National (mean)	19.00	28.00	
	Standard %	min.30%	min.30%	
Initiated a structured training program	NFS	92.59 (100/108)*	84.62 (143/169)	0.048
	HOL	100 (89/89)*	#	
	National (mean)	73.00	84.00	
	Standard %	min.80	min.80	
Readmitted within 4 weeks	NFS	4.51 (6/133)*	7.07 (14/198)	0.338
	HOL	10.19 (11/108)*	#	
	National (mean)	8.00	9.00	
	Standard %	max.10	max.10	

NFS and HOL compared to the national mean and the national standard: 2008 and 2012.

*Significant differences between hospitals compared in years (two proportions z-test).

#Data missing. HOL stopped reporting to the database in 2011.

### Bleeding and perforated ulcer

In patients admitted with a bleeding ulcer 2 of 5 indicators were significantly improved after the reorganization in NFS; “endoscopy within 24 hours” (60.00% vs. 84.16%, p = 0.005) and “endoscopy treatment of rebleeding” (40.00% vs. 100.00%, p = 0.018). In HOL, 2 of 5 indicators also improved significantly (both: p < 0.05) (Table 5).

**Table 5:** **Bleeding ulcer indicators**

**Indicator**	**Location**	**Pre percent (n) 2008**	**Post percent (n) 2012**	**p-value chi2-test**
Endoscopy within 24 hours from admission/time to decision about treatment	NFS	60.00 (18/30)*	84.16 (85/101)*	0.005
	HOL	90.00 (9/10)*	71.67 (43/60)*	0.219
	National (mean)	83.00	84.00	
	Standard %	min.85	min.85	
Treatment/therapeutic endoscopy,	NFS	66.67(6/9)	90.70 (39/43)	0.055
	HOL	100 (6/9)	88.24 (15/17)	0.379
	National (mean)	92.00	94.00	
	Standard %	min.90	min.90	
Rebleeding after primary treatment	NFS	16.67 (1/6)*	10.42 (5/48)	0.646
	HOL	66.67 (4/6)	9.52 (2/21)	0.003
	National (mean)	16.00	12.00	
	Standard %	max.15	max.15	
Endoscopic treatment of rebleeding	NFS	40.00 (2/5)	100 (7/7)*	0.018
	HOL	40.00 (2/5)	55.56 (5/9)*	0.577
	National (mean)	72.00	73.00	
	Standard %	min.75	min.75	
Surgical treatment of primary bleeding or rebleeding	NFS	10.00 (3/30)	4.81 (10/104)	0.290
	HOL	30.00 (3/10)	4.76 (3/63)	0.007
	National (mean)	5.00	4.00	
	Standard %	max.10	max.10	

NFS and HOL compared to the national mean and the national standard: 2008 and 2012.

*Significant differences between hospitals compared in years (two proportions z-test).

No significant improvements were seen in patients admitted with a perforated ulcer in NFS, consistent with results from HOL, although the indicator measuring “daily weight control” improved in HOL (33.33% vs. 100.00%, p = 0.007). Generally the numbers of patients in this section are low (Table 6).

**Table 6:** **Perforated ulcer indicators**

**Indicator**	**Location**	**Pre percent (n)**	**Post percent (n)**	**p-value chi2-test**
Operation time frame within 6 hours	NFS	66.67 (4/6)	93.75 (15/16)	0.099
	HOL	100 (4/4)	88.89 (8/9)	0.488
	National (mean)	61.00	61.00	
	Standard %	min.75	min.75	
Reoperation	NFS	33.33 (2/6)	31.25 (5/16)	0.926
	HOL	25.00 (1/4)	11.11 (1/9)	0.522
	National (mean)	16.00	16.00	
	Standard %	max.10	max.10	
Weight control (daily)	NFS	33.33 (2/6)	56.25 (9/16)*	0.338
	HOL	33.33 (1/3)	100 (9/9)*	0.007
	National (mean)	33.00	61.00	
	Standard %	min.90	min.90	
Fluid balance (daily)	NFS	66.67 (4/6)	75.00 (12/16)	0.696
	HOL	100 (4/4)	88.89 (8/9)	0.488
	National (mean)	72.00	86.00	
	Standard %	min.90	min.90	
Postoperative monitoring (daily)	NFS	66.67 (4/6)	93.75 (15/16)	0.099
	HOL	100 (4/4)	77.78 (7/9)	0.305
	National (mean)	69.00	93.00	
	Standard %	min.90	min.90	

NFS and HOL compared to the national mean and the national standard: 2008 and 2012.

*Significant differences between hospitals compared in years (two proportions z-test).

### Hip fracture

Both comparing hospitals showed significant improvements in the two indicators concerning hip fracture, comparing pre- and post-intervention measures (all: p < 0.006); except for the decreasing rehabilitation indicator in NFS in the same period (95.75% vs. 82.27%, p < 0.0001) (Table 7).

**Table 7:** **Hip fracture indicators**

**Indicator**	**Location**	**Pre percent (n) 2008**	**Post percent (n) 2012**	**p-value chi2-test**
Pain	NFS	65.89 (141/214)	85.88 (146/170)	0.000
	HOL	81.92 (222/271)	98.17 (161/164)	0.000
	National (mean)	#	88.00	
	Standard %	min.90	min.90	
Rehabilitation	NFS	95.75(203/212)	82.27(181/220)	0.000
	HOL	94.83 (257/271)	99.47 (187/188)	0.006
	National (mean)	#	93.00	
	Standard %	min.90	min.90	

NFS and HOL compared to the national mean and the national standard: 2008 and 2012.

#Data missing.

### Mortality

Significant reductions in the 30 day-mortality in patients admitted with stroke were seen when the pre- and the post-intervention data were compared for both NFS (12.29% vs. 5.66%, p = 0.012) and HOL (11.81% vs. 8.04%, p = 0.024). A significant reduction in 1 year mortality was also observed at NFS in patients with heart failure (44.71% vs. 15.10%, p < 0.0001). Despite small numbers of patients in that section, mortality due to bleeding ulcers was significant lower in NFS before the reorganization than after compared to HOL that showed significantly decreased mortality in the same period (Table 8).

**Table 8:** **Mortality**

**30-day Mortality**	**Locations**	**Pre percent (n) 2008**	**Post percent (n) 2012**	**p-value chi2-test**
Stroke	NFS	12.29 (36/293)	5.66 (12/212)	0.012
	HOL	11.81 (72/652)	8.04 (51/634)	0.024
	National (mean)	10.00	10.00	
	Standard %	max. 15	max. 15	
COPD	NFS	4.35 (3/69)	8.49 (45/530)	0.233
	HOL	6.61 (8/121)	10.76 (47/437)	0.176
	National (mean)	9.00	10.00	
	Standard %	None	None	
Heart failure **(1 year mortality)**	NFS	44.71 (60/136)*	15.1 (31/206)	0.000
	HOL	33.03 (36/109)*	#	
	National (mean)	#	13.00	
	Standard %	max.20	max.20	
Bleeding ulcer	NFS	3.45 (1/29)*	9.62 (10/104)	0.286
	HOL	20.00 (2/10)*	10.94 (7/64)	0.415
	National (mean)	9.00	9.00	
	Standard %	max.10	max.10	
Perforated ulcer	NFS	33.33 (2/6)	31.25 (5/16)	0.926
	HOL	25.00 (1/4)	37.50 (3/8)	0.665
	National (mean)	23.00	22.00	
	Standard %	max.20	max.20	
Hip fracture	NFS	11.68 (25/214)	8.47 (21/248)	0.251
	HOL	11.81 (32/271)	9.43 (20/212)	0.406
	Standard %	max 10	max 10	

NFS and HOL compared to the national mean and the national standard: 2008 and 2012.

*Significant differences between hospitals compared in years (two proportions z-test).

#Data missing.

After the establishment of the new ED in NFS 63% of indicators meet national standard compared to 30% before and in HOL 55% of the indicators meet national standards compared to 60% before.

## Discussion

This study aimed to investigate whether reorganization of EDs, with observation units and several interventions improve clinical quality of five specific diseases, evaluated with measures defined by Danish health care authorities [1]. The results indicate an overall improved clinical quality and a reduced mortality of patients with stroke, heart failure, bleeding ulcer and hip fracture after the establishment of the ED with observation units compared to baseline.

Overall, data shows a significant positive change in 53% of all the indicators in patients admitted in NFS and 46% of all the indicators in HOL. We argue that it could reflect patient safety issues and flow indicators in five common diseases seen in the ED. Internationally, other studies have demonstrated that the reorganization, in relation to the establishment of EDs, optimize patient safety as well as the flow of patients into and out of the ED [2,6,7,18,19]. Mortality decreased significantly in patients admitted with stroke in both hospitals and in patients admitted with heart failure in NFS. The mortality data are credible because they are crosschecked with mortality data from The Danish National Patient Registry (LPR). Other interventional studies show similar results on various types of indicators, e.g. mortality, readmissions and waiting time. Generally a consensus of indicators seems to be missing in literature. Our results supports international literature indicating that these improvements might originate from the implementation of the new concept for diagnosing and treating acute patients [3,9,20].

Several studies have shown that EDs with observation units and senior physicians in front might improve decision-making and workflow and demonstrates a more efficient use of hospital space. Patients are discharged earlier using fewer bed days and with readmission rates less than or equal to the first-time admission rates [10,21-23], indicating that the earlier discharge is not selling out on patient condition. Our results indicate a general decreased mortality in five of the disease groups in NFS; however, it includes a COPD group with unchanged or higher mortality in post data analyses, even though readmission rates shows consistent decrease in the same period. This could possibly be explained in the structure of the patient intake in the NFS ED. In the pre reorganization setup, very ill COPD patients with acute respiratory failure were directly admitted to the intensive care unit or directly in the COPD department and therefore not accounted for in the analyses. In the post reorganization setup, all patients, including the ones with respiratory failure, are seen in the ED, resulting in increasing mortality.

The nationally defined protocol for handling of patients with stroke has been used in the ED in NFS with a specific aim to implement standardized routines and increase competencies among staff in other care areas [24]. The use of the stroke protocol is seen in most other hospitals in Region Zealand. NFS faced a special challenge in 2009 since the results were significantly below the national standard as measured by the indicators for hip fracture. The results improved with the reorganization and they are similar in HOL as well as other hospitals nationwide [10]. The improvements, however, should not be attributed to the reorganization alone, as the general treatment has changed nationally over years including improved treatment protocols in some diseases and easier access to advanced treatment, e.g. fibrinolysis in stroke patients.

The two compared hospitals had important differences in their organization of the EDs. HOL already had its own group of senior and junior physicians in February 2009, whereas NFS was highly dependent on doctors from other departments to work in the ED; however, the observational beds were already established in 2009 in NFS compared to 2011 in HOL. Thus, the two hospitals faced different challenges in the reorganization period, emphasising the choice of the final comparison year as 2012.

To our knowledge this is the first study to investigate the quality of acute care in a representative group of acute conditions before and after in a reorganized ED over a longer period of time. Earlier studies in Denmark have focused on groups of specific diagnoses [25]. These studies are, however, done without consistency throughout studies and in various ways [26]. In the absence of a standard definition of quality, measuring clinical quality in an ED is complex and difficult and fosters the question of which indicators are the most representative of a specific clinical setting [26,27].

It might be argued that using these indicators as a surrogate marker of clinical quality is inadequate; however, the indicators are the measuring tool of national choice, making it necessary to relate to. As great differences in accessibilities to different paraclinical tests as well as various medical specialists exists between hospitals, it is important to compare similar hospitals. Great concerns have been raised from the peripheral hospitals that the national indicators reflect standard care in a university hospital setting, but not in a peripheral hospital. This emphasise the importance of the positive post reorganizational results achieved in NFS as a peripheral hospital.

Whether the improvements observed were due to the establishment of the ED or as a result of a general improved quality in treatment is questionable. However, it is likely that an increased focus on early intervention in the ED might have encouraged these results. Therefore, the study indicates that during the period of the establishment of an ED, it was possible to improve the clinical quality in selected services that reflect early diagnosis and treatment. The development of a common set of indicators for monitoring acute treatment at EDs in Denmark is recommended as the indicators available are not specific in terms of acute care and only covers a part of the spectrum needed. We call for a set of validated indicators reflecting the acute changes in patients within 24–48 hours of admission in an ED.

### Limitations

The use of the validated indicators increased the reproducibility; yet, it cannot be excluded that there may be a risk of information bias because of missing or incomplete data records because of the various physicians reporting data. In general, the data completeness in the databases is high both at the departmental and the national level, only with some insignificant differences [10].

The patient records are comprehensive at the national, regional and departmental levels, but they vary according to the different disease groups. Some issues concerning internal validity are acknowledged by the authors, as the patients’ records were reported by different specialist physicians.

We noted a considerable difference in morbidity and mortality among the disease groups, which is why we chose indicators within the three primary specialties of the hospital: medicine, orthopaedic surgery and general surgery.

HOL is a comparable hospital with the establishment of an ED in April 2009 and with approximately the same activity.

## Abbreviations

ED: Emergency Department
NIP: The National Indicator project
RKKP: Regions clinical quality development
NFS: Nykøbing Falster Hospital
HOL: Holbæk Hospital
